# Phosphoinositide Signaling and Mechanotransduction in Cardiovascular Biology and Disease

**DOI:** 10.3389/fcell.2020.595849

**Published:** 2020-12-14

**Authors:** Amanda Krajnik, Joseph A. Brazzo, Kalyanaraman Vaidyanathan, Tuhin Das, Javier Redondo-Muñoz, Yongho Bae

**Affiliations:** ^1^Department of Pathology and Anatomical Sciences, Jacobs School of Medicine and Biomedical Sciences, University at Buffalo, State University of New York, Buffalo, NY, United States; ^2^Cell Biology Program, Memorial Sloan Kettering Cancer Center, New York, NY, United States; ^3^Department of Molecular Biomedicine, Centro de Investigaciones Biológicas Margarita Salas, Madrid, Spain; ^4^Lydia Becker Institute of Immunology and Inflammation, Faculty of Biology, Medicine and Health, School of Biological Sciences, University of Manchester, Manchester, United Kingdom

**Keywords:** phosphoinositides, cardiovascular mechanotransduction, actin cytoskeleton, ion channel, focal adhesion, PIP_2_, PIP_3_, PI3K

## Abstract

Phosphoinositides, which are membrane-bound phospholipids, are critical signaling molecules located at the interface between the extracellular matrix, cell membrane, and cytoskeleton. Phosphoinositides are essential regulators of many biological and cellular processes, including but not limited to cell migration, proliferation, survival, and differentiation, as well as cytoskeletal rearrangements and actin dynamics. Over the years, a multitude of studies have uniquely implicated phosphoinositide signaling as being crucial in cardiovascular biology and a dominant force in the development of cardiovascular disease and its progression. Independently, the cellular transduction of mechanical forces or mechanotransduction in cardiovascular cells is widely accepted to be critical to their homeostasis and can drive aberrant cellular phenotypes and resultant cardiovascular disease. Given the versatility and diversity of phosphoinositide signaling in the cardiovascular system and the dominant regulation of cardiovascular cell functions by mechanotransduction, the molecular mechanistic overlap and extent to which these two major signaling modalities converge in cardiovascular cells remain unclear. In this review, we discuss and synthesize recent findings that rightfully connect phosphoinositide signaling to cellular mechanotransduction in the context of cardiovascular biology and disease, and we specifically focus on phosphatidylinositol-4,5-phosphate, phosphatidylinositol-4-phosphate 5-kinase, phosphatidylinositol-3,4,5-phosphate, and phosphatidylinositol 3-kinase. Throughout the review, we discuss how specific phosphoinositide subspecies have been shown to mediate biomechanically sensitive cytoskeletal remodeling in cardiovascular cells. Additionally, we discuss the direct interaction of phosphoinositides with mechanically sensitive membrane-bound ion channels in response to mechanical stimuli. Furthermore, we explore the role of phosphoinositide subspecies in association with critical downstream effectors of mechanical signaling in cardiovascular biology and disease.

## Introduction

**FIGURE 1:**
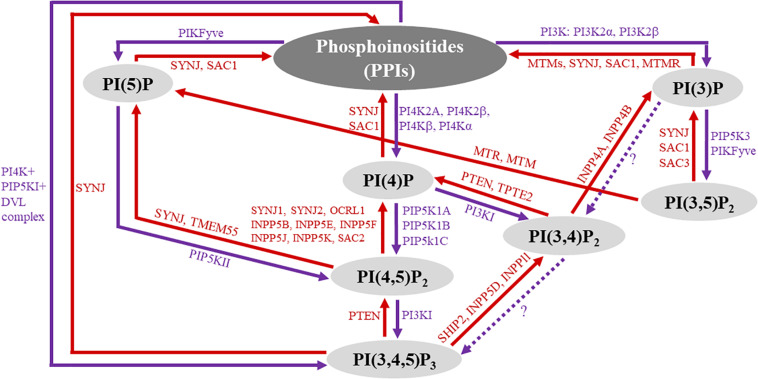
Overview of phosphoinositide (PPI) signaling. The diagram represents an overview of PPI subspecies and their biochemical interconversion. The seven biologically active PPI subspecies are highlighted in gray circles. PPI phosphatases are labeled red, and their associated reactions are represented by red arrows, indicating the direction of the reaction. Similarly, kinase reactions are represented by purple arrows with kinase enzymes labeled purple. Question marks along the dotted arrows represent areas that have yet to be explored.

Phosphoinositides (PPIs) constitute less than five percent of all cell membrane phospholipids (Hammond and Hong, 2018) but are essential to the integrity of all living cells (Dickson and Hille, 2019). Numerous studies have shown that PPIs are critical to cellular functions, including but not limited to cell proliferation, survival, motility, differentiation, and cytoskeletal dynamics (Di Paolo et al., 2004; Huang et al., 2004; Tsujita and Itoh, 2015; De Craene et al., 2017; Senju et al., 2017; Hao et al., 2018; Ramos et al., 2018; Bilanges et al., 2019; Li H. et al., 2019; Hirsch et al., 2020). The generation of PPIs is mediated by phosphorylation and dephosphorylation of phosphatidylinositol, the membrane lipid precursor (De Craene et al., 2017). More specifically, the inositol head of phosphatidylinositol can be phosphorylated at the 3-, 4-, and 5-hydroxyl positions of the inositol ring. The attachment of phosphate(s) can occur at any of these positions singularly or in combination to generate seven biologically active PPI subspecies: PI(3)P, PI(4)P, PI(5)P, PI(3,4)P_2_, PI(4,5)P_2_, PI(3,5)P_2_, and PI(3,4,5)P_3_ (Di Paolo and De Camilli, 2006; De Craene et al., 2017). All seven PPI subspecies naturally occur in the cell membrane of eukaryotes to varying degrees and are chemically interconverted by cell-specific kinases (purple-colored text in Figure 1) and phosphatases (red-colored text in Figure 1). PPI subspecies are shown as the black-colored text in Figure 1. Once biochemically active, PPIs modulate a tremendous breadth of horizontal and vertical cell signaling crosstalk spanning the cell membrane and cytoplasm, respectively, in which high-affinity interactions occur among various pleckstrin homology (PH) domain-containing membrane-based and cytosolic effector proteins, including protein kinase B (PKB)/Akt, protein kinase C (PKC), phosphoinositide phospholipase C (PLC), 3-phosphoinositide-dependent protein kinase-1 (PDK1), and small G proteins (Prestwich, 2004; Ghigo and Li, 2015; Manna and Jain, 2015; De Craene et al., 2017). In the cardiovascular system, activated PPI signaling mediates enzymatic organic modification of secondary messenger proteins because PPIs are crucial scaffolding proteins to complex signalosomes of cardiac and vascular cellular functions, and their aberration is a prominent driving force in cardiovascular pathology (Falkenburger et al., 2010; Ghigo and Li, 2015; Schink et al., 2016).

Mechanotransduction describes the physiological process by which cells perceive and respond to mechanical stimuli, including tensile stretch and compression, shear stress, and extracellular matrix (ECM) stiffness. Moreover, mechanical cues are converted into intracellular biochemical signals in which the resultant cytoskeletal and nuclear remodeling modulates cellular functions (Tschumperlin, 2011; Maurer and Lammerding, 2019). Mechanotransduction is vital to cardiovascular tissue development, growth, and homeostasis because cells are continuously under mechanical stress (Garoffolo and Pesce, 2019). Dysregulation of the mechanical harmony between the cell and ECM can drive the development and progression of pathology, including but not limited to cardiac ischemia and fibrosis, hypertension, and atherosclerosis (Gimbrone and Garcia-Cardena, 2013; Yue et al., 2015; Schafer et al., 2017; Ochoa et al., 2018; Russo et al., 2018). Only recently have we begun to understand the cellular mechanisms that mediate the signal transduction of mechanical stimuli, which greatly overlap with canonical biochemical cellular signaling pathways.

Today, cardiovascular disease (CVD) remains the leading cause of death and morbidity worldwide. A great majority of biomedical research in CVD centers around the known mechanisms of biochemical and molecular biology modalities. With the recent emergence of novel biomechanical and cell biological technologies and techniques, there has been a new integrative movement toward understanding the mechanical regulation of cellular biochemistry and molecular biology inside the cell. This review will span the most recent findings in phosphoinositide biology as it relates to mechanically sensitive cellular processes in cardiovascular cells in both homeostasis and disease. We will discuss how specific PPI subspecies mediate cytoskeletal remodeling processes known to be dominantly regulated by mechanotransduction and the direct interaction of PPIs with membrane-bound channels in response to mechanical stress in cardiovascular cells. Furthermore, we will explore the role of PPI subspecies in association with the essential effectors of mechanical signaling in cardiovascular biology and disease.

## Phosphatidylinositol-4,5-Bisphosphate (PIP_2_)

### PIP_2_ Association With Actin Cytoskeleton Dynamics

**FIGURE 2:**
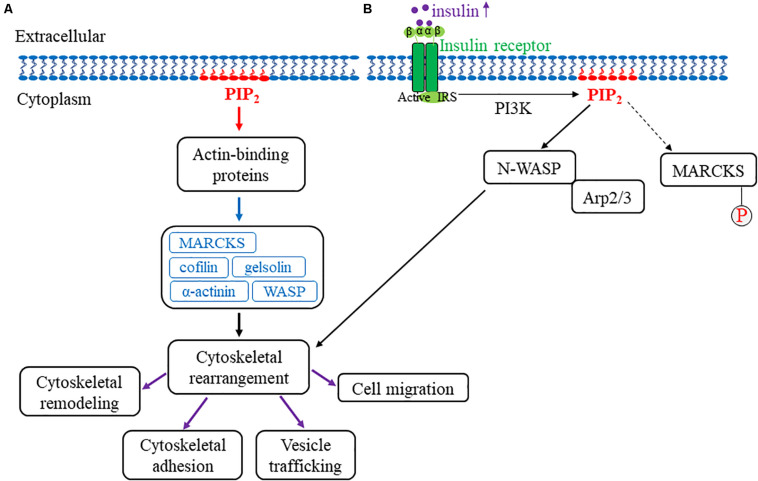
Model for PIP_2_ association with the actin cytoskeleton. **(A)** An overview of PIP_2_’s role in actin cytoskeletal remodeling. The red arrow represents PIP_2_ intracellular association with actin-binding proteins. The blue arrow represents the various PIP_2_-associated actin-binding proteins, including MARCKS, cofilin, WASP, gelsolin, and α-actinin. Upon interaction with PIP_2_, these proteins collectively mediate cellular cytoskeletal rearrangement (indicated by the black arrow). More specifically, actin cytoskeletal remodeling, cytoskeletal adhesion, vesicle trafficking, and cell migration are associated with cytoskeletal rearrangements that are mediated by PIP_2_ interaction with actin-binding proteins and are indicated by the purple arrows. **(B)** This figure depicts how PIP_2_ regulates cytoskeletal rearrangement through N-WASP and Arp2/3 with MARCKS phosphorylation. Once the insulin receptor is activated, insulin increases intracellularly and subsequently mediates the dissociation of MARCKS from PIP_2_ (as indicated by the dotted arrow) in which MARCKS is subsequently phosphorylated and activated. In addition, PIP_2_ separately binds to and activates both N-WASP and Arp2/3, which drives actin cytoskeletal rearrangement. Together, these findings highlight the role of PIP_2_ in mechanosensitive actin cytoskeletal rearrangement and remodeling.

The actin cytoskeleton is a complex and dynamic intracellular structure that gives mechanical rigor to the cell while simultaneously mediating the transduction of mechanical stress into biochemical signals. Given the unique contractile properties of cardiac and vascular cells, the actin cytoskeleton is most essential to their integrity (Allahverdian et al., 2018; Zhang et al., 2018). Alteration of cytoskeletal organization, specifically actin filament dynamics, can result in gene expression and cell proliferation modification with the subsequent adaptation of and changes to intracellular biochemical responses and cellular functions, respectively. Thus, cytoskeletal remodeling can mediate mechanical stress-induced gene expression, cell proliferation, and pathological processes (Ohashi et al., 2017; Martino et al., 2018). Recent studies have shown that PIPs regulate cytoskeletal arrangement and signaling dynamics (Di Paolo and De Camilli, 2006; Saarikangas et al., 2010; Senju and Lappalainen, 2019). Specifically, PIP_2_ is involved in cytoskeletal reorganizational events, including vesicle trafficking, cell migration, phagocytosis, and membrane cytoskeletal adhesion (Saarikangas et al., 2010; Shewan et al., 2011; Dickson and Hille, 2019; Phan et al., 2019). PIP_2_ binds to and affects actin-binding proteins, such as myristoylated alanine-rich C kinase substrate (MARCKS), cofilin, gelsolin, α-actinin, Wiskott-Aldrich syndrome protein (WASP), and the Rho family of small GTPases (Janmey et al., 2018; Figure 2). MARCKS is an actin-binding protein found in mammalian tissues and, upon phosphorylation, it binds reversibly to structural and regulatory molecules in the cell in which there is an associated decrease in PIP_2_ binding (Nairn and Aderem, 1992; Sheetz et al., 2006). Additionally, in vascular endothelial cells (ECs), MARCKS directly modulates PIP_2_-mediated insulin signaling. The treatment of vascular ECs with insulin increases the levels of PIP_2_, which is also released into lipid rafts (caveolar and non-caveolar fractions) to bind to the cytoskeletal protein, N-WASP. Subsequently, N-WASP phosphorylation and interaction with actin-related proteins 2/3 (Arp2/3) cause cytoskeletal remodeling to induce cell migration (Kalwa and Michel, 2011). Furthermore, MARCKS mediates a PIP_2_-dependent actin rearrangement process. In this process, when low levels of MARCKS are present, actin filaments form an actin gel. Conversely, actin filaments aggregate upon increased levels of MARCKS. In the plasma membrane, the overall PIP_2_ levels are relatively constant; however, changes in PIP_2_ levels can be observed locally in the membrane directly overlying actin protrusions and membrane ruffles (Sheetz et al., 2006).

Rac, a downstream small GTPase effector, is a regulator of membrane ruffles (Ridley, 1994). In addition, Rac is instrumental to the transduction of external mechanical stimuli (Labouesse, 2011; Lawson and Burridge, 2014; McGowan and McCoy, 2017), including the mechanotransduction of the FAK-Cas-Rac axis, which transmits ECM stiffness into intracellular stiffness and mechanosensitive cell cycling (Bae et al., 2014). Moreover, PIP_2_ levels fluctuate in membrane ruffles in a Rac-dependent manner. Although the immediate relationship of PIP_2_ and Rac has not been explored in the context of cardiovascular biology and disease specifically, acknowledging their possible connection could better aid the understanding of their effects in different cellular pathways and help implicate PIP_2_ as a Rac-mediated effector in vascular pathology (Polacheck et al., 2017; Narumiya and Thumkeo, 2018).

### PIP_2_ Association With CapZ and Mechanical Stiffness

**FIGURE 3:**
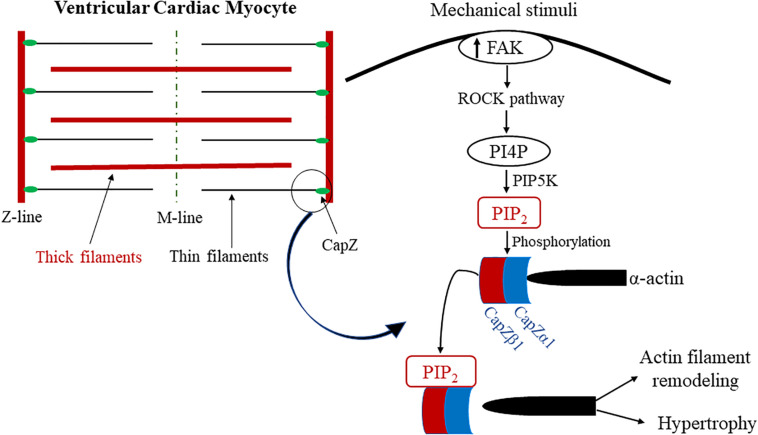
Proposed model of PIP_2_ and CapZ mechanotransduction in cardiac myocytes. Diagram of the proposed model for the mechanism of PIP_2_ association with CapZ because of mechanical stimuli. Mechanical strain activates the ROCK pathway when there is an increase in mechanosensitive FAK activation, which results in PIP5K phosphorylating PI4P into PIP_2_. PIP_2_ phosphorylates CapZ, which mediates its uncapping from α-actin. This results in pathological actin filament remodeling and cardiac hypertrophy. PIP_2_ is highlighted in and colored red. CapZα1 and CapZβ1 are colored blue to represent their association with PIP_2_ as well as their intricacy with biomechanically sensitive actin filament remodeling and pathological cardiac hypertrophy.

In striated muscle, capping protein Z (CapZ), an actin-capping protein, regulates cytoskeletal remodeling (Edwards et al., 2014). CapZ’s relationship with PIP_2_ (Figure 3) has been recently observed in relation to mechanical stiffness and cytoskeletal remodeling, and the PIP_2_-mediated interaction with CapZ has been shown to regulate cardiac myocyte hypertrophy (Li et al., 2016) and actin dynamics (Li and Russell, 2013). More specifically, ECM stiffening induces cardiac myocyte hypertrophy by increased PIP_2_ localization at the sarcomere Z-discs in cardiac myocytes (Li et al., 2016). The Z-disc is a critical site for mechanotransduction and the location of the β1-isoform of CapZ (CapZβ1) (Russell et al., 2010). Moreover, the localization of PIP_2_ to the sarcomere Z-disc is crucial to ventricular cardiac myocyte mechanotransduction and associated with pathological hypertrophic remodeling. Dysregulation of PIP_2_ signaling alters sarcomere integrity by modulating the function of CapZβ1 and actin dynamics. Taken together, PIP_2_ is vital to cardiac cell physiology, where it regulates CapZβ1 and actin dynamics in response to mechanical stimuli. Additionally, mechanical stimulation causes the production of PIP_2_, specifically through the RhoA/Rho-associated kinase (ROCK) pathway (Li and Russell, 2013). Solís et al. explored PIP_2_ signaling effects on CapZ through neomycin, a PIP_2_ sequestering agent, in neonatal ventricular cardiomyocytes cultured on varying substrate stiffnesses. Further studies have assessed the molecular mechanisms by which different mechanotransduction signaling pathways mediate the capping and uncapping of CapZ from actin filaments via PIP_2_. The results showed that interactions between PIP_2_ and the β-tentacle of CapZ after molecular stimulation become considerably modified by phosphorylation. Moreover, CapZ is bound tightly to actin when inactive; however, upon phosphorylation and activation in growth states of hypertrophy, the binding is loosened. This is triggered by external stimuli, including mechanical flexing, loading, a stiffer substrate, angiotensin II, and phenylephrine. CapZ is modified by the stimuli’s signaling pathways through phosphorylation, acetylation or PIP_2_ binding. Thus, an actin assembly mechanism can be presented where phosphorylation, acetylation or PIP_2_ anchorage causes CapZ to act as a nodal terminus for the integration of various signaling pathways (Solis and Russell, 2019). This mechanism implicates PIP_2_ as a critical mediator of mechanotransduction in cardiac myocytes by directly affecting CapZ in response to mechanical stiffness.

### PIP_2_, PLC, and PKC Association With Store-Operated Channels TRPC1/3/6 and Orai 1

Phospholipase C (PLC) is a critical membrane-associated enzyme that, when stimulated by Gαq/11 subtype protein-coupled receptors (GαqPCRs), catalyzes the hydrolysis of PIP_2_ phosphodiester bonds to generate inositol 1,4,5-triphosphate (IP_3_) and diacylglycerol (DAG), which further activates PKC. These secondary messengers and downstream effector proteins are important for cardiovascular cell functions because they orchestrate the regulation of intracellular calcium mobilization, which is critical not only to the contractile apparatus but also to cell survival, proliferation, and differentiation. Plasma membrane store-operated channels (SOCs), particularly transient receptor potential canonical channels 1, 3, and 6 (TRPC1/3/6) and calcium release-activated calcium (CRAC), also called Orai 1, 2, and 3, are activated as a consequence of PLC activation (Abdullaev et al., 2008; Baudel et al., 2020; Wang et al., 2020). Vascular smooth muscle cell (VSMC) contraction, proliferation, and migration are regulated by the stimulation of SOCs at the plasma membrane and their associated Ca^2+^ influx pathways (Baudel et al., 2020). Moreover, these cellular behaviors are associated with the development of diseases of the vasculature, such as hypertension and atherosclerosis (Baudel et al., 2020). In VSMCs, PKC activity and PIP_2_ are important in the activation pathway of SOCs, particularly transient receptor potential canonical channel 1 (TRPC1) (Saleh et al., 2009b; Shi et al., 2012; Baudel et al., 2020). Upon Ca^2+^ store depletion, TRPC1 is phosphorylated by PKC, which itself is stimulated by the PLC-PIP_2_-DAG pathway (Saleh et al., 2009a), thus establishing a potentially direct link between TRPC1 and PIP_2_. Additionally, TRPC1 is known to be an essential component of various mechanotransduction pathways, specifically in cells where TRPC1 is crucial for mechanosensitive cell migration (Formigli et al., 2009; Garrison et al., 2012; Canales et al., 2019; Li et al., 2019). Interestingly, TRPC1 is upregulated in pathological neointima remodeling in vessels induced by vascular injury, further suggesting that the induction of these channels is mechanosensitive (Kumar et al., 2006). Recent findings by Nikolaev et al. have suggested that the TRP ion channel superfamily is involved in a wide variety of mechanosensory processes, yet it has been shown that such channels are insensitive to tension induced by cell membrane stretching. Thus, although several TRP channels, including TRPC1, are essential components of mammalian stretch-activated mechanosensitive calcium-permeable cation channel heterologous systems, their true role in mechanotransduction remains unclear (Gottlieb et al., 2008). It is quite plausible that these ion channels are more likely to be activated by upstream components and consequently act as amplifiers of cellular mechanosensory signaling cascades, including PLC and PKC (Nikolaev et al., 2019). In addition to TRPC, the Orai channel, or CRAC, is another class of Ca^2+^-selective SOC activated as a consequence of PLC activation and subsequent PIP_2_ depletion (Abdullaev et al., 2008; Baudel et al., 2020; Wang et al., 2020). This channel is expressed in VSMCs and upregulated in such cells in vascular pathologies, including vascular injury and restenosis, which are known to be mechanically mediated (Wang et al., 2008; Spinelli and Trebak, 2016). Previous reports have shown that Orai interacts with TRPC channel subtypes, including TRPC3 and TRPC6 (Liao et al., 2007); however, TRPC 1 is independent of Orai function (DeHaven et al., 2009; Shi et al., 2017). Although these two proteins share great similarity in their functionality, it has yet to be explored how they may be coupled mechanically. Previous studies have assessed the mechanosensitivity of the Orai channel; however, it remains to be empirically determined (Dong et al., 2019). Furthermore, Piezo1 is a recently characterized putative mechanically activated calcium permeable cation channel that is ubiquitously expressed through the cardiovascular system (Beech and Kalli, 2019). It has been uniquely shown that Piezo1’s mechanosensitivity to membrane tension is regulated by PIP_2_ levels. Upon activation of TRPV1, PLC is activated and depletes the local levels of PIP_2_, which subsequently inhibits Piezo1’s mechanosensitive activity (Borbiro et al., 2015).

### PIP_2_ in Mechanotransduction of Capillary Signaling

The capillary endothelial cell (cEC) inward rectifier K^+^ channel Kir2.1 is critical to sensing and translating neural activity and neurovascular coupling in brain cECs (Longden and Nelson, 2015). This process of capillary-to-arteriole signaling in cECs is regulated by PIP_2_. Moreover, PIP_2_ levels are required for sustained Kir2.1 activity, and such regulation of Kir2.1 channels mediates electrical signaling during neurovascular coupling (Harraz et al., 2018a). More specifically, GαqPCRs stimulate PLC to rapidly either deplete or decrease PIP_2_ levels and subsequently suppresses Kir2.1 channel signaling (Harraz et al., 2018a). This depletion of PIP_2_ also promotes the activation of transient receptor potential vanilloid 4 (TRPV4), a channel found in cECs that is inhibited by PIP_2_ under basal conditions and because of GαqPCR activation (Harraz et al., 2018b). Furthermore, PIP_2_ levels govern capillary-to-arteriole electrical signaling by modulating the activity of TRPV4 and Kir2.1, which regulate the cellular states of depolarization and hyperpolarization. Thus, the levels of PIP_2_ considerably modulate the magnitude of electrical signaling across cerebral capillaries, which ultimately affects cerebral microcirculatory blood flow in cECs (Harraz et al., 2018a,b). The relationship of TRPV4 to PIP_2_ is important given TRPV4’s involvement in shear stress mechanotransduction in endothelial cells and mesenchymal cells and its ability to act mechanosensitively (Kohler and Hoyer, 2007; Yin and Kuebler, 2010; Corrigan et al., 2018). Therefore, the role of PIP_2_ in this signaling pathway and its interaction with a known mechanotransducer, TRPV4, suggests that PIP_2_ itself acts in the mechanotransduction of capillary electrical signaling.

## Phosphatidylinositol-4-Phosphate 5-Kinase (PIP5K)

### PIP5K Association in Focal Adhesion and the Cytoskeleton

Phosphatidylinositol-4-phosphate 5-kinase (PIP5K) phosphorylates the fifth position of the inositol head of phosphatidylinositol-4-phosphate. The type 1 PIP5K subfamily comprises three isoforms, Iα, Iβ, and Iγ, and is critical to many cytoskeletal processes. It has been reported that the overexpression of these isoforms induces the formation of stress fibers, membrane ruffles, and microvilli and regulates actin cytoskeletal dynamics, suggesting that this enzyme and its PIP_2_ products are mechanosensitive (Chatah and Abrams, 2001). Weernink et al. reported that RhoA and its kinase, ROCK, which are both dominant effectors of mechanotransduction, are essential regulators of PIP5K in HEK-293 cells. The overexpression of ROCK enhances the PIP5K activity and subsequently elevates PIP_2_ formation. Conversely, the chemical inhibition of ROCK decreases PIP5K activity and PIP_2_ formation (Oude Weernink et al., 2000). Furthermore, Weernink et al. examined Type 1 PIP5K through other Rho family small GTPases, including Rac1 and Cdc42. Rho GTPases, RhoA, Rac1, and Cdc42 all mediate the PIP5K levels and lead to an increase in PIP_2_ levels (Weernink et al., 2004). Therefore, PIP5K activity is RhoA-dependent in which signals from RhoA to the actin cytoskeleton are mediated, and synthesis of PIP_2_ is enhanced (Oude Weernink et al., 2000).

The PIP_2_ synthesis pathway in platelets through the isoform PIP5K Iα was more closely studied by Chatah and Abrams (2001) and Trepat et al. (2005). Thrombin, a known mediator of actin cytoskeleton remodeling (Chatah and Abrams, 2001; Trepat et al., 2005), promotes PIP_2_ synthesis by PIP5K from PI4P in response to G protein-coupled receptor stimulation. PIP5K Iα localizes in the Golgi under basal conditions. Following stimulation of PAR1, a thrombin receptor, or overexpression of the active variant of Gα_*q*_, PIP5K Iα relocates to the plasma membrane. This translocation of PIP5K Iα is dependent on Rac1 and RhoA. Rac1 has been suggested to affect PIP5K indirectly, and activation is required by Rho (Chatah and Abrams, 2001).

Although these studies independently identified Rho GTPases in mediating PIP5K activity, the mechanisms by which Rho GTPase is suggested to activate PIP5K are separate. Taken together, these findings indicate that members of the Rho GTPase family, RhoA, Rac, and Cdc42, are vital in mediating PIP5K activation and, consequently, PIP_2_ synthesis, regardless of their interconversional crosstalk (Oude Weernink et al., 2000; Chatah and Abrams, 2001; Weernink et al., 2004). Furthermore, these GTPases act as a dynamic molecular switch between various cells, which play a key role in vascular pathology (Cai et al., 2015; Karoor et al., 2018; Barlow and Cleaver, 2019) and are involved in mechanosensing and mechanotransduction pathways (Verma et al., 2011; Chaterji et al., 2014; Zegers and Friedl, 2014; Ohashi et al., 2017). The relationship of PIP5K with small downstream GTPases in vascular pathology and mechanotransduction has not yet been explored. Due to the relevance of the small GTPases Rho, Rac, and Cdc42 for PIP5K activity and PIP_2_ synthesis, this pathway may be vitally important for better understanding vascular disease and may be potentially significant in the overall study of mechanotransduction in the context of vascular pathology. Therefore, the relationship of these PIP5Ks and these downstream GTPases should be explored in relation to mechanotransduction and vascular disease.

## Phosphoinositide 3-Kinase (PI3K) and Phosphatidylinositol-3,4,5-Triphosphate (PIP_3_)

### Akt/PI3K Signaling in the Mechanotransduction of Ventricular Cardiomyocytes

Phosphoinositide 3-kinase (PI3K) is a family of evolutionarily conserved lipid kinases that mediate many cellular responses to physiological and pathophysiological stimuli. The PI3K family is divided into three subgroups (classes I, II, and III), which together include eight isoforms. The class I isoforms, PI3Kα, PI3Kβ, PI3Kγ, and PI3Kδ, convert PIP_2_ to phosphatidylinositol-3,4,5-triphosphate (PIP_3_) (Vanhaesebroeck et al., 2010; Miller et al., 2019). Activated PI3K produces PIP_3_, which further recruits 3-phosphoinositide-dependent kinase 1 (PDK1) to the plasma membrane (Hagiwara et al., 2012). PIP_3_ activates PDK1 through its PH domain. PDK1 subsequently phosphorylates and activates Akt at threonine residue 308 (T308) (Ghigo and Li, 2015; Manning and Toker, 2017). More importantly, the phosphorylation of serine residue 473 (S473) by the mechanistic target of the mammalian target of rapamycin complex 2 (mTORC2) stabilizes not only T308 phosphorylation but also AKT in its active state (Manning and Toker, 2017). Together, Akt and PI3K create a unique signaling pathway (Akt/PI3K) that is instrumental in cardiomyocyte mechanotransduction (Li C.J. et al., 2019). Moreover, the Akt/PI3K signaling pathway regulates intracellular and extracellular activities in response to mechanical stress and molecular effectors, leading to a robust cellular mechanotransduction signaling cascade in cardiac myocytes. These cellular responses include modulation of cell metabolism, growth, proliferation, angiogenesis, and cardiac adaptation (Aoyagi and Matsui, 2011; Markowska et al., 2014; Yang et al., 2018). In a disease model, chronic activation of the Akt/PI3K pathway dysregulates cell contractility, which induces compensatory cardiac hypertrophy with preserved contractility and ultimately advances to chronic dilated cardiomyopathy (Shiojima et al., 2005; Li C.J. et al., 2019). Furthermore, alterations in the function and structure of titin, a giant sarcomeric filament protein, have been observed in similar cardiomyopathies, including cardiac remodeling, hypertrophy, and heart failure (Linke, 2008; Kruger and Linke, 2009; Lyon et al., 2015). In cardiac sarcomeres, titin isoforms exhibit varying properties of mechanical elasticity and are differentially expressed throughout cardiac development and during disease in which isoform switching is dynamically regulated by the Akt/PI3K signaling pathway (Kruger and Linke, 2009). Moreover, it is believed that these properties of titin are uniquely positioned to serve as a molecular sensor of mechanical stress in cardiac myocytes, including oscillatory changes in cell stretching known to induce PI3K activation through molecular mechanisms that remain unclear (Miller et al., 2004; Linke, 2008; Leychenko et al., 2011; Voelkel and Linke, 2011).

### PIP_3_, PI3Kα, and PI3Kγ Association With Mechanotransduction Through Gelsolin and Cyclic Adenosine Monophosphate (cAMP)

In cardiac myocytes, mechanotransduction critically mediates remodeling of the cytoskeleton, and dysregulation of this process can drive heart disease in response to aberrant biomechanical stress. Biomedical research on patients with hypertension has revealed how critical cardiac mechanotransduction plays in this response (Patel et al., 2013). One study by Patel et al. demonstrated that PI3Kα, a major PI3K isoform in the heart, negatively regulates gelsolin activity and suppresses pathological cytoskeletal remodeling in response to biomechanical stress-induced cardiac mechanotransduction and the resulting dilated cardiomyopathy (Guo et al., 2010). Similarly, a separate study showed that loss of PTEN in ventricular cardiac myocytes increases PI3Kα activity, which attenuates pressure overload-induced heart failure but loss of myocardial contractility (Oudit et al., 2008). Conversely, however, other studies have shown that constitutively activated PI3K drives the growth and hypertrophy of such cells, greatly increasing the heart size in mice, while knocking down PI3Kα results in mice with smaller hearts (Shioi et al., 2000). In response to mechanical stress, PI3Kα translocates to the plasma membrane to convert PIP_2_ to PIP_3_, which subsequently recruits gelsolin to the plasma membrane (Patel et al., 2018). A resulting spatial colocalization occurs between p110α, the catalytic subunit of PI3Kα, and gelsolin in which p110α-catalyzed PIP_3_ negatively regulates gelsolin activity and thus diminishes unfavorable remodeling of the actin cytoskeleton while conserving the cytoskeletal integrity. Consequently, PI3Kα-generated PIP_3_ plays a critical role in the mechanotransduction of cardiomyocytes by negatively regulating gelsolin, which subsequently inhibits actin remodeling (Patel et al., 2018).

In cardiac myocytes, GPCRs activate PI3Kγ in response to pressure overload or biomechanical stress, which mediates the adaptive role in cardiac mechanotransduction by negatively regulating cyclic adenosine monophosphate (cAMP) levels (Guo et al., 2010). It was first shown that complete deletion of PI3Kγ in cardiac myocytes alters heart function by inducing cell hypercontractility as a result of cAMP accumulation but does not alter the cell structure or growth (Crackower et al., 2002; Patrucco et al., 2004); however, a separate study has shown that deletion of PI3Kγ accelerates the development of pathological hypertrophy (Guo et al., 2010). Intriguingly, the regulation of cell contractility by PI3Kγ in response to mechanical stress is independent of its activity or functional kinase domain (Patrucco et al., 2004). More specifically, cardiac myocytes lacking PI3Kγ activity with preserved expression exhibit normal levels of cAMP that are believed to be the result of phosphodiesterase 3B positive regulation by a PI3Kγ-associated multifunctional protein complex (Patrucco et al., 2004). Critical to this complex is the anchoring of PKA to PI3Kγ and downstream activation of phosphodiesterases, type 3 and 4 (PDE3/4), and subsequently reducing the cAMP levels; upon its anchoring, PKA also phosphorylates and inhibits PI3Kγ lipid kinase activity, resulting in a reduction in PIP_3_ (Perino et al., 2011; Ghigo et al., 2017). In pressure overload-mediated sympathetic overdrive of cardiac myocytes, the beta2 adrenergic receptor is desensitized and internalized as a result of PKA-escaped PI3Kγ kinase activity and ultimately induces hypokinetic dilated heart failure (Prasad et al., 2005; Perino et al., 2011; Ghigo and Li, 2015; Ghigo et al., 2017). Despite enhanced calcium dynamics and contractility upon the loss of PI3Kγ in cardiac myocytes, decompensation ensues because of dysregulated cellular-ECM interactions (Guo et al., 2010). Furthermore, a more direct relationship between PI3Kγ and cardiac mechanotransduction is observed upon the loss of PI3Kγ, in which elevated cAMP levels mediate extracellular matrix remodeling and interactions (Guo et al., 2010). In this particular instance, inhibiting the beta2 adrenergic receptor protects N-cadherin adhesion complexes from degradation (Guo et al., 2010), whereas the loss of p110γ function, the catalytic subunit of PI3Kγ, leads to heart failure by the deterioration of N-cadherin and an increase in cAMP levels (Patel et al., 2018). Furthermore, N-cadherin complexes actively perceive biomechanical stress, and through the regulation of gelsolin, actin polymerization is promoted, therefore expressing a collaborative relationship between PI3Kγ and PI3Kα in cardiac mechanotransduction (Chan et al., 2004).

### PI3K Mechanotransduction Association With the Hippo Pathway Through YAP/TAZ

The Hippo signaling pathway, which was originally observed in Drosophila, mediates the VSMC stretch response that inhibits cell proliferation and participates in mechanotransduction pathways (Huang et al., 2005; Ota and Sasaki, 2008; Yu et al., 2015; Chakraborty et al., 2017; Fletcher et al., 2018). Inhibition of the Hippo pathway promotes tissue growth in epithelial cells through the PI3K-PDK1-Akt axis upon mechanical stimulation and growth factor signaling (Borreguero-Munoz et al., 2019). Yes-associated protein 1 (YAP) and transcriptional coactivator with the PDZ-binding motif (TAZ) are downstream transcriptional activators of the Hippo pathway (Halder et al., 2012). These effectors are regulated by mechanical cues, specifically, matrix stiffness, stretch, and cell density, which influence cell proliferation and differentiation (Halder et al., 2012; Codelia et al., 2014; Meng et al., 2016). Thus, YAP and TAZ function as essential effectors of mechanotransduction (Meng et al., 2018). YAP/TAZ-dependent glutaminolysis and anaplerosis are mechanoactivated by vascular stiffness to drive cell proliferation in pulmonary hypertension (Bertero et al., 2016). Additionally, mechanical stretching regulates YAP/TAZ activity via the PI3K-PDK1-mediated pathway in human umbilical arterial VSMCs (Wang et al., 2018). Furthermore, the PDK1 interaction with the Hippo complex is mediated through Sav1, where PDK1 directly controls the Hippo pathway (Wang et al., 2018). The consequential association of PI3K with the Hippo signaling pathway effectors YAP and TAZ in vascular cells further implicates PI3K in the mechanotransduction of the cardiovascular system.

### PI3K Implication in Mechanotransduction of Vascular Remodeling

Mechanical forces of a hemodynamic nature are uniquely fundamental for vascular homeostasis as well as pathological vascular remodeling that are commonly observed in CVD (Cahill and Redmond, 2016; Russo et al., 2018). In cells of the vasculature, harmony in cell proliferation, apoptosis, migration, and differentiation is integral to vascular wall homeostasis. Mechanical forces perceived by ECs and VSMCs generate a biological response, i.e., mechanotransduction to induce physiological vascular remodeling (Qi et al., 2018). Consequently, vascular remodeling involves a variety of cellular components to mediate these biophysical and biochemical events, including PI3K, which has previously been connected to the vascular remodeling pathway. During angiogenesis, vessel remodeling can help with cell proliferation and maturation (Wang and Khalil, 2018). Vascular remodeling in pericytes is regulated by PI3Kβ. Mature pericytes, which are mostly found in vessels undergoing remodeling, are quiescent and express low activation of the PI3K signaling. Inactivation of PI3Kβ in these cells generates early pericyte maturation, with an increase in PI3K signaling that obstructs pericyte maturation. Thus, pericytes in a sustained immature state will result in vascular hyperplasia and block vascular remodeling, whereas accurate PI3K signaling is necessary for pericyte maturation and correct vessel formation (Figueiredo et al., 2020).

One of the most prevalent cardiovascular diseases involving vascular remodeling is atherosclerosis. During atherosclerosis, vascular injury occurs, causing abnormal proliferation of VSMCs, which leads to neointima formation and vessel lumen narrowing and ultimately limits blood flow and oxygen supply (Yu et al., 2018). PI3K has been directly associated with the molecular pathways that mediate vascular remodeling and atherosclerosis. The catalytic subunit of PI3Kα, p110α, is important for receptor tyrosine kinase (RTK) signaling, which is upstream of class 1A PI3K isoforms, in VSMCs. Furthermore, p110α is critical to neointima formation after balloon angioplasty by mediating VSMC proliferation and migration, while the PI3Kα isoforms p100β and p110δ do not play a significant role (Vantler et al., 2015).

PI3Kγ functions in both leukocytes and cardiomyocytes and plays a role in atherosclerosis and heart disease. PI3Kγ controls leukocyte infiltration in the myocardium and arteries. PI3Kγ is involved in neuraminidase-1 (Neu-1) signaling, which governs atherosclerosis development (Gayral et al., 2014). Genetic and pharmacological inhibitory targeting of PI3Kγ in leukocytes reduces atherosclerosis in mouse models (Fougerat et al., 2008). Ghigo et al. (2017) recently reviewed PI3K and calcium signaling in cardiovascular disease. The PI3K pathway has recently been interconnected with Ca^2+^ signaling. PI3Kγ appears to be preferentially linked to Ca^2+^ signaling in smooth muscle cells (Lupieri et al., 2020), where Class I PI3Ks are highly expressed. This interconnection between the PI3Kγ pathway and Ca^2+^ signaling has been involved in smooth muscle cell proliferation and migration, atherosclerosis and arterial injury. The development of arterial lesions through various immune functions requires PI3Kγ activity with PI3Kγ playing an important role in arterial injury in T cells. For example, it has been found that PI3Kγ regulates T-cell function, and it has been proposed that PI3Kγ interacts with Ca^2+^ signaling, leading to Ca^2+^ influx downstream of T-cell receptor activation; thus, PI3Kγ interconnects with Ca^2+^, playing an important role in arterial injury (Smirnova et al., 2014; Lupieri et al., 2015; Ghigo et al., 2017). Taken together, PI3Kβ and PI3Kγ are paramount pathways that drive cardiovascular remodeling seen in heart failure as well as in atherosclerosis, and this strongly suggests that PI3K is critically involved in mechanotransduction-mediated cardiovascular disease.

## Conclusion

This review summarizes the relationship between PPIs and mechanotransduction in regard to cardiovascular biology and disease (Table 1). PPIs are central mediators in multiple biological processes, although understanding the specific contribution of PPIs to cellular dynamics can be difficult, especially regarding mechanotransduction in cardiovascular disease. PIP_2_, PIP_3_, PI3K, and PIP5K all play important roles in different mechanotransduction pathways of the cardiovascular system. These PPI functions include cytoskeletal arrangements, association with actin-binding proteins and ion channels, and response to mechanical stimuli. Indeed, PPIs are critical modulators of mechanotransduction. Complete knowledge of these pathways is not yet fully known and should be further explored to address how these pathways influence cellular mechanotransduction in cardiovascular cells in both homeostasis and disease.

**TABLE 1:** Overview of phosphoinositide signaling and mechanotransduction in cardiovascular biology and pathology.

**PPIs**	**Associated protein(s)**	**Cell type(s)**	**Known related function(s)**	**Proposed mediation in mechanotransduction**
**PIP_2_**	**MARCKS**	**vECs** (Kalwa and Michel, 2011)	Cytoskeletal rearrangement: - PIP_2_ and MARCKS interaction observed in membrane ruffles of which Rac is a regulator. - PIP_2_ levels fluctuate in membrane ruffles in a Rac-dependent manner-indicating a possible relationship between Rac and PIP_2_ (Kalwa and Michel, 2011)	Rac involved in numerous mechanotransduction pathways (i.e., FAK-Cas-Rac axis) (Labouesse, 2011; Lawson and Burridge, 2014; McGowan and McCoy, 2017)
**PIP_2_**	**CapZ**	**Ventricular CMs** (Solis and Russell, 2019)	PIP_2_ acts as a mechanical sensor at sarcomere Z-disc in response to mechanical stimuli (Solis and Russell, 2019)	Sarcomere Z-disc located on CapZβ1 is a site for mechanotransduction (Russell et al., 2010)
**PIP_2_ and PKC**	**TRPC1**	**VSMCs** (Saleh et al., 2009b; Shi et al., 2012; Baudel et al., 2020)	Functions are associated with the development of vascular diseases (Saleh et al., 2009b; Shi et al., 2012; Baudel et al., 2020)	TRPC1 is implicated in mechanotransduction (Formigli et al., 2009; Garrison et al., 2012; Canales et al., 2019; Li N. et al., 2019)
**PIP_2_**	**Kir2.1 and TRPV4**	**Cerebral capillary ECs** (Harraz et al., 2018a,b)	PIP_2_ controls the capillary-to-arteriole electrical signaling through depolarization or hyperpolarization of TRPV4 and Kir2.1 (Harraz et al., 2018a,b)	TRPV4 has been observed in shear stress-mediated mechanotransduction in ECs and mesenchymal cells (Kohler and Hoyer, 2007; Yin and Kuebler, 2010; Corrigan et al., 2018)
**PI3K**	**Akt**	**Ventricular CMs** (Aoyagi and Matsui, 2011; Markowska et al., 2014; Yang et al., 2018)	Akt/PI3K signaling pathway regulates cellular functions in response to mechanical stress, including cell metabolism, growth, proliferation, angiogenesis, and cardiac adaptation (Aoyagi and Matsui, 2011; Markowska et al., 2014; Yang et al., 2018)	The Akt/PI3K signaling is a known mediator of mechanotransduction in ventricular CMs (Li et al., 2019)
**PI3Kα**	**Gelsolin**	**Ventricular CMs** (Guo et al., 2010)	PI3Kα regulates gelsolin activity (Guo et al., 2010)	PI3Kα plays a role in biomechanical stress-induced ventricular CM mechanotransduction (Guo et al., 2010)
**PI3Kγ**	**cAMP**	**Ventricular CMs** (Guo et al., 2010)	In response to biomechanical stress, G protein-coupled receptors activate PI3Kγ, and thus negatively regulate cAMP (Guo et al., 2010)	PI3Kγ plays a role in ventricular CM mechanotransduction (Guo et al., 2010)
**PI3K**	**PTEN**	**Ventricular CMs** (Shioi et al., 2000; Luo et al., 2005)	Overexpression of PTEN reduces the levels of PI3K and influences the growth and the hypertrophy of ventricular cardiomyocytes (Shioi et al., 2000; Luo et al., 2005)	
**PI3Kα**	**Gelsolin and p110α**	**Ventricular CMs** (Patel et al., 2018)	PI3Kα translocates and induces the spatial colocalization between p110α and gelsolin, resulting in the attenuation of actin cytoskeleton remodeling (Patel et al., 2018)	PI3Kα-generated PIP_3_ plays a critical role in the mechanotransduction through gelsolin (Patel et al., 2018)
**PI3K**	**Hippo pathway through YAP/TAZ**	**Epithelial cells** (Borreguero-Munoz et al., 2019)	Inhibition of the Hippo signaling pathway promotes tissue growth via PI3K-PDK1-Akt axis (Borreguero-Munoz et al., 2019)	YAP and TAZ are essential effectors of mechanotransduction and effectors of mechanical cues (Halder et al., 2012; Codelia et al., 2014; Meng et al., 2016)
**PI3Kγ**	**cAMP, N-cadherin and gelsolin**	**Ventricular CMs** (Guo et al., 2010)	- Reduction of N-cadherin and an increase in cAMP levels result in the loss of p110γ function, which can lead to heart failure. - Actin polymerization is promoted through gelsolin in response to biomechanical stress (Guo et al., 2010)	PI3Kγ plays a role in ventricular CM mechanotransduction (Chan et al., 2004)
**PI3K**	**Hippo pathway through YAP/TAZ**	**Human umbilical arterial SMCs** (Wang et al., 2018)	Mechanical cell stretching regulates YAP/TAZ activity *via* PI3K/PDK1-mediated pathway (Wang et al., 2018)	YAP and TAZ are essential effectors of mechanotransduction and effectors of mechanical cues (Halder et al., 2012; Codelia et al., 2014; Meng et al., 2016)
**PI3Kβ**	**RGS (Regulator of G protein signaling 5)**	**Pericytes** (Figueiredo et al., 2020)	Accurate PI3K signaling is necessary for pericyte maturation and correct vessel formation (Figueiredo et al., 2020)	Mechanotransduction induces physiological vascular remodeling (Qi et al., 2018)
**PI3Kα**	**Receptor tyrosine kinase**	**VSMCs** (Vantler et al., 2015)	Catalytic subunit of PI3Kα, p110α, is essential to pathological neointima formation (Vantler et al., 2015)	Mechanotransduction induces pathological vascular remodeling in atherosclerosis (Yu et al., 2018)
**PI3Kγ**	**Elastin-derived peptides and GPCR kinase-2**	**Leukocytes and CMs** (Fougerat et al., 2008)	- PI3Kγ is involved in Neu-1 signaling which governs atherosclerosis development (Gayral et al., 2014) - Genetic and chemical inhibition of PI3Kγ reduces atherosclerosis *in vivo* (Fougerat et al., 2008) - PI3Kγ directly interacts with GPCR kinase-2 which is observed in cardiac failure (Ghigo and Li, 2015)	Mechanotransduction induces pathological vascular remodeling in atherosclerosis (Yu et al., 2018)
PIP5K*	**RhoA and ROCK**	**HEK-293** (Weernink et al., 2004)	- RhoA, Rac1, and Cdc42 regulate cellular PIP5K levels. - PIP5K activity is RhoA-dependent in which signals from RhoA to the actin cytoskeletal mediate enhanced PIP_2_ synthesis (Weernink et al., 2004)	RhoA, Rac, and Cdc42 are mediators of mechanotransduction (Verma et al., 2011; Chaterji et al., 2014; Zegers and Friedl, 2014; Ohashi et al., 2017)
PIP5K*	**Thrombin, Rac, and Rho**	**HEK 293 and Cos-7** (Chatah and Abrams, 2001; Trepat et al., 2005)	- Thrombin promotes PIP_2_ synthesis and separately relocates PIP5K Iα to the plasma membrane. - Translocation of PIP5K Iα is dependent on Rac1 and RhoA; Rac1 is suggested to effect PIP5K indirectly and activation is required by RhoA (Chatah and Abrams, 2001; Trepat et al., 2005)	Rho GTPase family, RhoA, Rac, and Cdc42 are known mechanosensors (Verma et al., 2011; Chaterji et al., 2014; Zegers and Friedl, 2014; Ohashi et al., 2017)

This table summarizes all the main phosphoinositide (PPI) subspecies, in the context of cardiovascular pathology and biology, which are assessed in our review with their main associations and the cellular target(s) of these interactions. The main function from these associations and how the PPIs are connected to mechanotransduction or as mechanosensors are further highlighted in this table. *These studies express interconversional crosstalk. Although regardless of their crosstalk these results indicate RhoA, Rac, and Cdc42 are essential in mediating PIP5K activation. vECs, Vascular endothelial cells; VSMCs, Vascular smooth muscle cells; CMs, Cardiomyocytes.

## Author Contributions

AK, JB, and YB conceptualized the review. AK, JB, KV, TD, and YB wrote the original draft. AK and JB prepared the figures and table. AK, JB, KV, TD, JR-M, and YB critically reviewed and edited the final manuscript version. All authors contributed to the article and approved the submitted version.

## Conflict of Interest

The authors declare that the research was conducted in the absence of any commercial or financial relationships that could be construed as a potential conflict of interest.
